# Identification, Evolution and Expression of an Insulin-Like Peptide in the Cephalochordate *Branchiostoma lanceolatum*

**DOI:** 10.1371/journal.pone.0119461

**Published:** 2015-03-16

**Authors:** Claire Lecroisey, Yann Le Pétillon, Hector Escriva, Eckhard Lammert, Vincent Laudet

**Affiliations:** 1 Molecular Zoology Team, Institut de Génomique Fonctionnelle de Lyon, École Normale Supérieure de Lyon, CNRS, Université Lyon, Lyon, France; 2 CNRS, UMR 7232, BIOM, Observatoire Océanologique, F-66650 Banyuls/Mer, France; 3 Institute of Metabolic Physiology, Heinrich-Heine University Düsseldorf, 40225 Düsseldorf, Germany; Academia Sinica, TAIWAN

## Abstract

Insulin is one of the most studied proteins since it is central to the regulation of carbohydrate and fat metabolism in vertebrates and its expression and release are disturbed in diabetes, the most frequent human metabolic disease worldwide. However, the evolution of the function of the insulin protein family is still unclear. In this study, we present a phylogenetic and developmental analysis of the Insulin Like Peptide (ILP) in the cephalochordate amphioxus. We identified an ILP in the European amphioxus *Branchiostoma lanceolatum* that displays structural characteristics of both vertebrate insulin and Insulin-like Growth Factors (IGFs). Our phylogenetic analysis revealed that amphioxus ILP represents the sister group of both vertebrate insulin and IGF proteins. We also characterized both temporal and spatial expression of ILP in amphioxus. We show that *ilp* is highly expressed in endoderm and paraxial mesoderm during development, and mainly expressed in the gut of both the developing embryo and adult. We hypothesize that ILP has critical implications in both developmental processes and metabolism and could display IGF- and insulin-like functions in amphioxus supporting the idea of a common ancestral protein.

## Introduction

**Fig 1:**
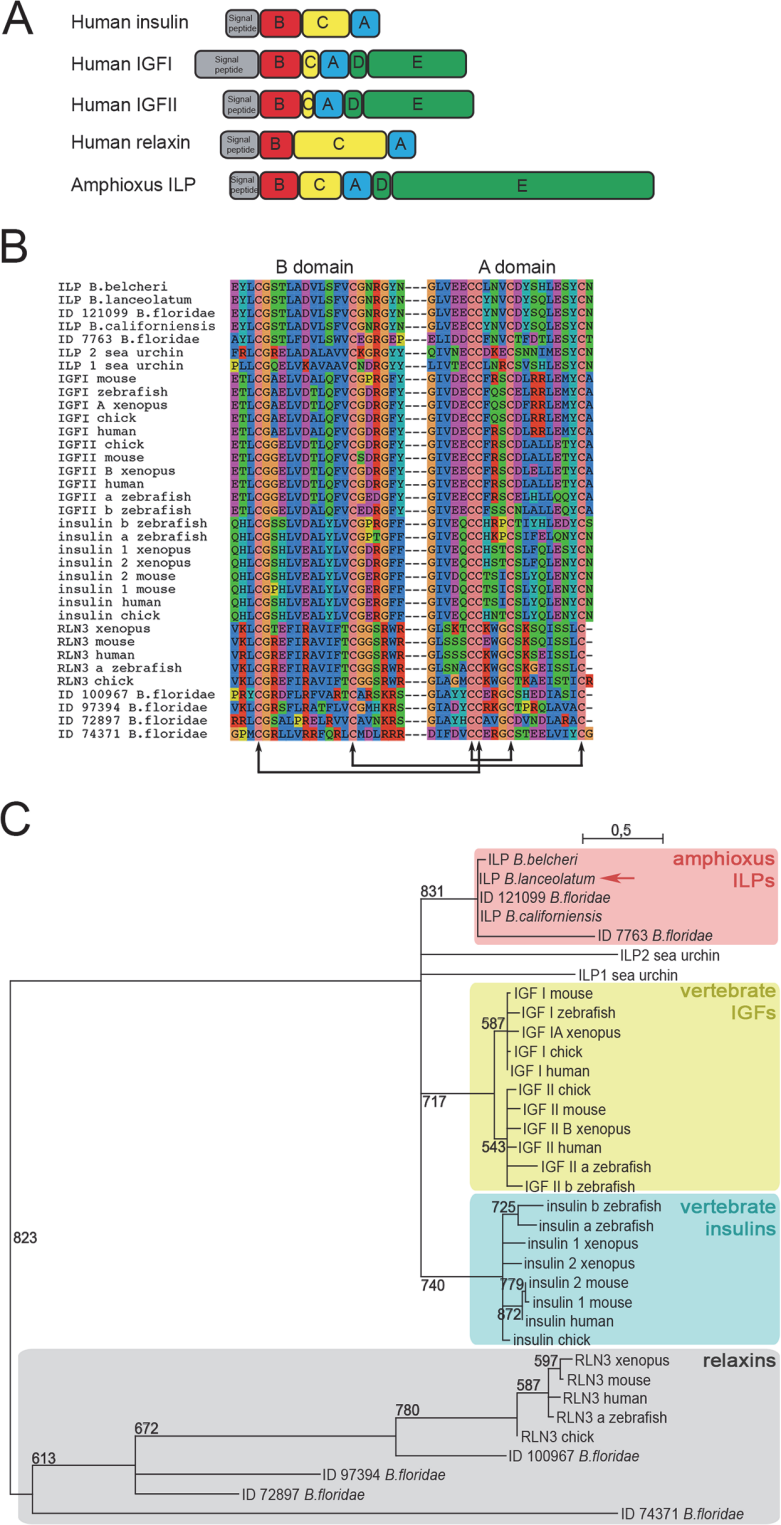
Structural comparison and phylogenetic relationships between amphioxus ILP and insulin-relaxin superfamily proteins in vertebrates. A. Comparison of amphioxus ILP domain structure with human insulin, IGFs and relaxin. B. Sequence alignment of the conserved A and B domains of ILP sequences from different amphioxus species and insulin, IGF and relaxin sequences from vertebrates and sea urchin. Black arrows indicate the conserved cysteine residues necessary for the formation of disulfide bonds during the processing of precursor proteins. C. Phylogenetic maximum likelihood analysis of chordate insulin/IGF subfamily members. The red square highlights amphioxus ILP sequences, the blue square highlights vertebrates insulin sequences and the yellow square highlights vertebrates IGF sequences; the grey square indicates the outgroup made with relaxin sequences. *B*. *lanceolatum* ILP sequence identified in this study is pointed with the red arrow. Bootstrap values derived from 1000 runs are shown. Branches with bootstraps lower than 500 were collapsed. The scale bar indicates the average number of amino acid substitutions per site.

Proteins of the insulin-relaxin superfamily are implicated in critical physiological processes like metabolism, growth control, reproduction, cardiovascular function, and longevity in a wide range of animals. The insulin-relaxin protein family is characterized by the presence of a cystein knot motif. It consists of two subfamilies, the insulin and insulin-like growth factors (IGFs) subfamily and the relaxins (RLN) and insulin-like (INSL) subfamily [1]. These proteins exhibit a high degree of structural conservation, sharing the conserved cysteine residues required for the formation of disulfide bridges, which are the hallmark of this superfamily (Fig. 1A and B). Moreover, they share the same modular organisation of their precursor, including an N-terminal signal peptide followed by three domains (i.e. A, B and C domains). Processing the precursor proteins usually removes the N-terminal signal peptide, the C-peptide is cleaved by endoproteolytic enzymes, and the remaining peptides A and B are covalently linked by 3 disulfide bonds to give rise to the mature protein [1]. In contrast, IGF proteins are slightly different from other insulin-relaxin superfamily members. IGF holds additional D and E domains at the C terminus. The A domain is terminally extended and the C domain is not removed during the processing of the IGF precursor [2].

The origin of the insulin-relaxin superfamily has been a longstanding question. The similarity between IGF and insulin protein sequences has lead to the hypothesis of a common phylogenetic origin [1,3,4].

Insulin-like peptides (ILPs) are an ancient protein family and two sequences have been identified in the genome of the sea anemone *Nematostella vectensis* [5]. In protostomes, many ILPs arose from multiple independent lineage-specific gene duplications. In insects, ILPs were first identified as bombyxins, a multigenic family from the silkmoth *Bombyx mori*, and subsequently identified in the migratory locust, mosquitos and fruit fly among others [6,7]. Seven ILPs were also identified in molluscs from the snail *Lymnea stagnalis* [6,8]. In the nematode, ILPs constitute a multigenic family of forty members [9]. Neurosecretory cells mainly produce ILPs in insects and molluscs [7,8] and in *C*. *elegans*, characterized ILPs are mainly expressed in neurons [9]. In protostomes ILPs have key roles in metabolism, growth, reproduction, and aging [6,7,9]. It is important to note that ILP sequences from protostomes are described as insulin/IGF homologues, and no relaxin/INSL orthologous sequences have yet been identified in protostomes [10,11].

In deuterostomes, two sequences have been identified in the ascidian *Chelyosoma productum* as members of the insulin-relaxin superfamily on the basis of sequence and expression analysis. However, their phylogenetic positions has not been solved because of their high divergence and it has been speculated that these genes are the result of a recent lineage-specific gene duplication [4]. Three sequences have also been identified in the ascidian *Ciona intestinalis*. As with *Chelyosoma*, their orthology relationships with vertebrate IGFs and insulin is still not clear due to short sequences without sufficient number of informative sites [12].

Recently, two ILPs (SpILP1 and SpILP2) have been characterized in the sea urchin *Strongylocentrotus purpuratus* [13]. The gene structure of SpILP1 is similar to an insulin-like protein while the gene structure of SpILP2 is more divergent. The study suggests that SpILP1 and SpILP2 most probably arose from a sea urchin specific independent gene duplication. SpILP2 mRNA is mostly expressed in the gut of sea urchin embryo while SpILP1 mRNA is expressed in the stomach and in the intestine of the feeding larvae. It has been suggested that SpILP2 could behave as a growth signal during embryogenesis and SpILP1 could be involved in feeding related processes [13].

In the cephalochordate amphioxus, an *ilp* gene was first identified in *Branchiostoma californiensis* as a single copy gene [3]. An orthologous sequence was also identified in *B*. *floridae* [14] and in *B*. *belcheri* but the authors called this protein BbIGF instead of ILP [15]. Moreover, the sequencing of the *B*. *floridae* genome revealed 6 putative members of the insulin-relaxin superfamily [16]. Analyses of these sequences revealed that two of them (protein IDs 121099 and 77763) are related to the insulin/IGF subfamily [17], but the phylogenetic positions of the other four sequences have not been investigated.

Amphioxus ILP sequences display structural characteristics of both insulin and IGF sequences from vertebrates: sequence analyses suggest that amphioxus ILP is proteolytically cleaved like proinsulin, releasing the C-terminal peptide but it also contains extended D and E domains as found in vertebrate IGFs [3,15,18]. It has been shown that in adult *B*. *belcheri*, *ilp* (named BbIGF in this study) is mainly expressed in the hindgut and in the hepatic caecum, which is the putative liver precursor [15]. However, none of the amphioxus *ilp* genes identified previously have been characterized with spatio-temporal expression including development.

In this study, we identified the first ILP from the European amphioxus *Branchiostoma lanceolatum*. We analysed the phylogenetic relationship between all the insulin-like sequences that have been previously identified in amphioxus and their relationship with other proteins from the insulin-relaxin superfamily in chordates. We also present the first characterization of amphioxus *ilp* spatio-temporal expression both by qPCR and *in situ* hybridization. We show that *ilp* is first expressed in mesoderm, before being required in the endoderm and in the developing gut of the amphioxus embryo. We also show by qPCR that *ilp* is highly expressed in the gut in the adult amphioxus.

## Results and Discussion

### The amphioxus ILP is closely related to both insulin and IGF from vertebrates

The ILP protein sequence from *B*. *californiensis* was used as a template for *in silico* identification of the *B*. *lanceolatum ilp* using the reference transcriptome [19]. Then, we used oligonucleotides designed against the predicted *B*. *lanceolatum ilp* sequence for RT-PCR. RT-PCR were performed using cDNA synthesized from total RNA from adult amphioxus and a full length ORF encoding a complete ILP (309aa) was cloned.

As previously described for *B*. *californiensis* and *B*. *belcheri*, ILP protein sequence from *B*. *lanceolatum* presents structural characteristics of both insulin and IGF (Fig. 1A and B) [3,15]. *B*. *lanceolatum* ILP shows 88,8% sequence identity with ILP from *B*. *californiensis*, 90,8% sequence identity with ILP sequence from *B*. *belcheri* (named BbIGF) and 84,1% sequence identity with sequence ID 121099 from *B*. *floridae* genome, but only 26,9%, 17,1%, 15,7%, 12,2% and 8,1% sequence identity with the 5 others sequences from *B*. *floridae* genome (respectively ID 77763, 100967, 72897, 74371 and 97394) [16].

We used phylogenetic tree reconstruction on conserved A and B domains to assess the evolutionary relationships between ILPs from different amphioxus species and members of the insulin/IGF subfamily from vertebrates using relaxin sequences as outgroup. The phylogenetic tree was built using maximum likelihood with 1000 bootstrap replicates (Fig. 1C and S1 Fig.). We observed three robust main branches: (i) amphioxus ILPs, (ii) vertebrate insulins; and (iii) vertebrate IGFs.

These results show that ILP sequence identified in *B*. *lanceolatum* is orthologous to the previously identified ILPs in *B*. *californiensis* [3], *B*. *belcheri* [15] and in *B*. *floridae* (ID 121099 [16]). This tree, along with sequence alignment, shows that the *B*. *floridae* sequence ID 77763 is the most divergent amphioxus ILP, an observation that is consistent with its divergent structure since it does not present the D and E domains and lacks some critical residues necessary for the proper folding of the protein (Fig. 1A, B and C). Further studies should be performed to determine if this gene is a divergent paralogue, a prediction artefact or a pseudogene.

Interestingly, our analysis reveals that the other *B*. *floridae* sequences previously identified as putative insulin-relaxin superfamily members (sequence ID 100967, 72897, 74371 and 97394 [16]) branch with vertebrate relaxins and not with the insulin/IGF subfamily. This is the first report of relaxin-like proteins in amphioxus.

This phylogenetic analysis shows that ILP sequences from amphioxus consist of a well-supported group close to both vertebrate insulin and IGFs. Of note, relationships between these three branches are still unclear since their grouping is not supported by a high bootstrap value, and we observed that vertebrate IGFs and insulins are clustered together but with a low bootstrap value (236, S1 Fig.). This is why we present our tree as a polytomy (Fig. 1C). If we take into account the evolutionary history of vertebrates through two rounds of whole genome duplication [20], the polytomy presented in Fig. 1C suggests that amphioxus ILPs represent in fact the sister group (that is, an unduplicated version) of both the vertebrate insulins and vertebrate IGFs. Interestingly, amphioxus ILPs show structural and folding characteristics of both insulin and IGF [1,18] suggesting that subfunctionalization may have occurred after duplication in vertebrates even if functional data are still missing to substantiate this point.

### Spatio-temporal expression of *ilp* from *B*. *lanceolatum*

**Fig 2:**
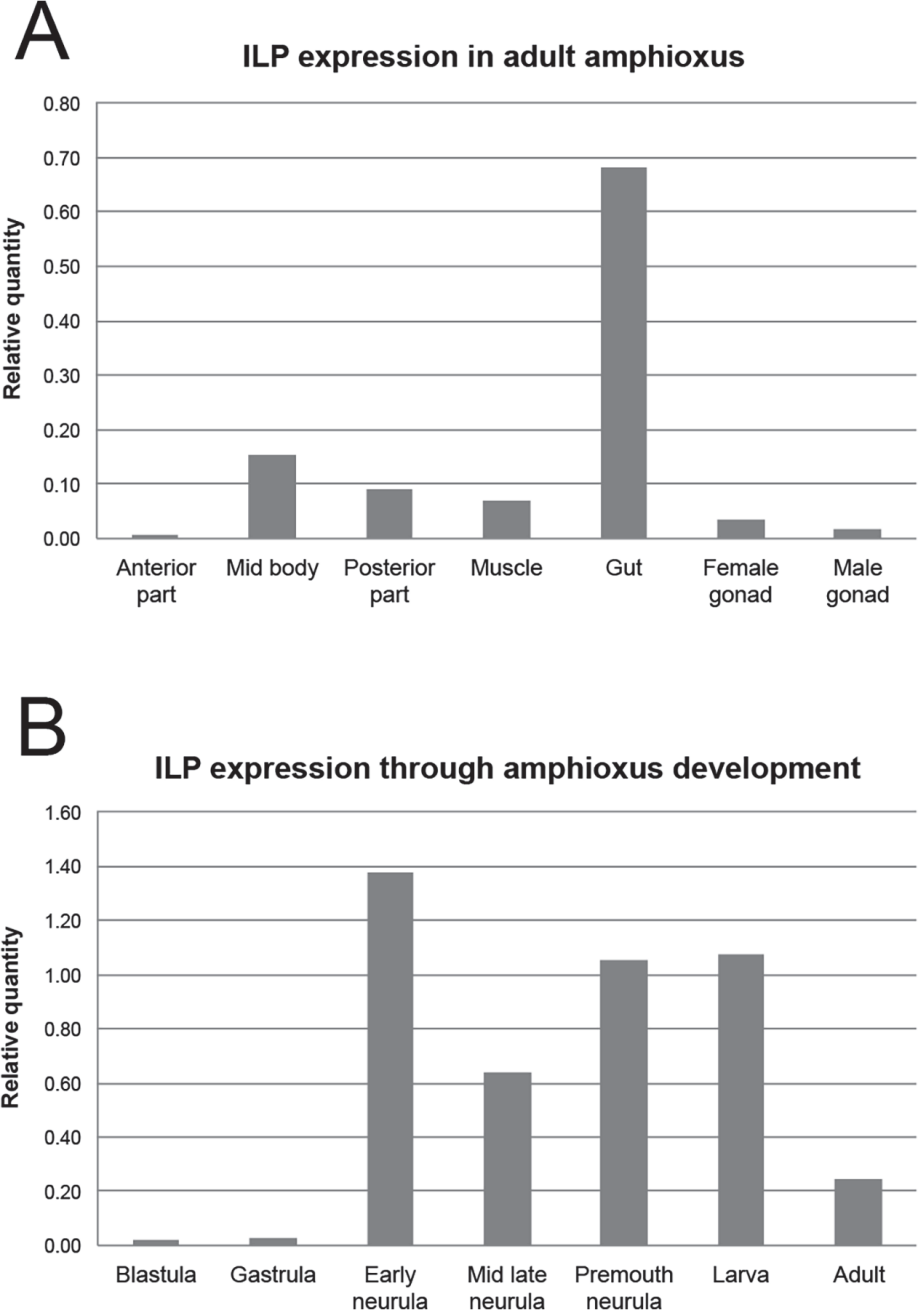
Expression analyses of *ilp* in adult amphioxus and during embryonic development. Expression level of amphioxus *ilp* in the adult (A) and during development (B). y-axis represents relative amount of *ilp* normalized with three different stable genes. Biological sample n = 1 in experiments presented in (A) and (B).

In order to characterize the expression of amphioxus *ilp* in the adult, we dissected mature animals and extracted RNA from the anterior, central and posterior parts, as well as muscle, gut and male and female gonads. Then, we analysed the level of *ilp* expression using a qPCR approach. Our results show that *ilp* is strongly expressed in the adult gut (Fig. 2A). This result is consistent with previous studies reporting expression of *ilp* in the amphioxus gut [15,21,22].

We then analysed *ilp* expression during amphioxus development. To do so, we extracted RNA at different developmental stages and in the adult (central part of the body, that contains the gut) to use as template for RT-PCR. Our results show a low level of expression during early development, then an increase of *ilp* expression at early-neurula stage and then the *ilp* expression is maintained until the larval stage (Fig. 2B).

**Fig 3:**
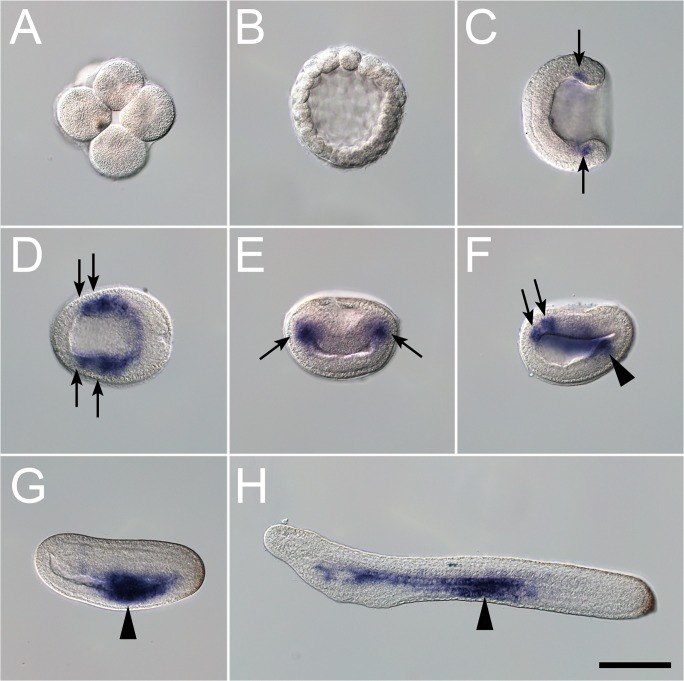
Expression pattern of amphioxus *ilp* during early embryogenesis. During the first developmental stages, (A) the eight cells embryos and (B) blastula stages, *ilp* is not expressed. (C) Dorsal view of a gastrula stage embryo, black arrows indicate *ilp* expression in the presumptive paraxial mesoderm. Dorsal (D), transversal (E) and left side (F) views of an early neurula embryo. Arrows show *ilp* signal in the paraxial mesoderm, the arrowhead shows the expression in the posterior endoderm. (G) Left side of a mid-neurula stage embryo, arrowhead shows *ilp* expression in the developing endoderm. (H) Left side of a late neurula stage embryo before the mouth opening. *ilp* is expressed in the anterior and central part of the endoderm, arrowhead shows the strongest signal in the mid-gut. Scale bars are 100μm. Anterior is to the left in C, D, F, G and H.

**Fig 4:**
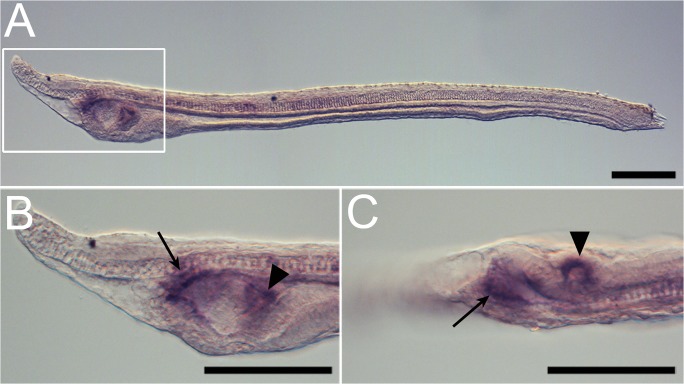
Expression pattern of amphioxus *ilp* in the larvae. (A) Left side of the larvae. (B) Enlargement of the pharyngeal region of the larvae shown in A. (C) Ventral view of the same larva. Arrowheads show *ilp* expression in the club shaped gland and arrows show *ilp* expression in the anterior pharyngeal endoderm. Scale bars are 100μm.

Amphioxus *ilp* expression during development was also visualized using whole mount *in situ* hybridization. *ilp* is first expressed at the gastrula stage in a restricted region corresponding to the future paraxial mesoderm (Fig. 3C). At the early neurula stage, *ilp* signal is still present in the paraxial mesoderm and it starts to be expressed in the posterior endoderm (Fig. 3D, E and F). Later, at the mid-neurula stage, the mesodermic expression disappears and the endodermic expression becomes conspicuous (Fig. 3G). This endodermic expression is then restricted to the pharyngeal as well as the mid-gut regions of the late neurula (before the mouth opening) (Fig. 3H). In the larva, *ilp* expression becomes restricted to the pharynx, mostly in the anterior pharyngeal endoderm and in the club shaped gland (Fig. 4). The club shaped gland is a transient structure that disappears at metamorphosis of the larva, and this gland exports secretions to the pharyngeal lumen most probably to help capture food particles [23].

In protostomes, ILPs are mainly localized in the nervous system (locust, drosophila, mosquito) where they function as neurohormones [6]. In amphioxus, we did not observe an expression pattern reminiscent of such a function for ILP. It is important to note that in insects, brain cells are the exclusive source of gastrointestinal hormones [24]. However, expression in the embryonic mid-gut have also been reported for drosophila DILP4 [25] and for some insulin-like genes (*ins*) in *C*. *elegans* [26] (http://www.wormbase.org).

In vertebrates, starting from early development, insulin expression becomes restricted to the pancreas where it is later produced by mature pancreatic beta cells [27,28]. However, insulin gene expression has also been reported in the central nervous system and liver [29]. IGFs are more ubiquitously expressed both in early embryo and in the adult, such as in the liver, brain, bone, ovary and testis [30].

As for vertebrates and sea urchins, *ilp* expression in amphioxus starts during early development. It is detected in endodermal tissues during development and later the majority of *ilp* is expressed in the gut which is consistent with the sea urchin *ilp2* expression in endodermal tissues in the embryo and *ilp1* expression in the gut of the larva [13]. The amphioxus *ilp* expression pattern could also be reminiscent of vertebrate expression of insulin in the pancreas and IGFs in the liver. It has been shown that in amphioxus, entero-endocrine cells do not form a cluster but are dispersed in the mid-gut epithelium and the pharynx [21,24]. The amphioxus mid-gut also has an extension named hepatic caecum which is considered the homologue of the vertebrate liver [31, 32]. Guo et al., 2009, demonstrated that BbIGF is expressed in the hepatic caecum and in the hind-gut of adult amphioxus, and our expression analyses also corroborate this observation. Interestingly, it has been shown in sea urchin that *ilp1* expression is food-dependent [13], and since *ilp* expression in endodermal derivatives (pharynx and gut) is concomitant with the mouth opening in amphioxus, it is tempting to suggest that ILP function may have a link with digestive functions.

However, the expression of *ilp* in the mesoderm during amphioxus development is quite unlike that of most other chordates. This transient developmental expression points to an early requirement of ILP specifically in paraxial mesoderm that has not been characterized in vertebrates. This difference may be correlated with the fact that amphioxus and vertebrates diverged during their evolutionary history for the formation of the anterior mesoderm. While amphioxus paraxial mesoderm is completely segmented from the most anterior to the most posterior part of the body, in vertebrates both anterior segmentation and anterior somites were lost during evolution and new mechanisms evolved secondarily to develop the head muscles [33]. Thus, the expression pattern of *ilp* in the anterior paraxial mesoderm of amphioxus may reflect an ancestral function that was lost in vertebrates during evolution. Later during amphioxus development, we detect no or very weak expression in gonads and muscles. Taken altogether, these results suggest that ILP plays an early function during development in mesodermal embryonic tissues that is specific to amphioxus and might recapitulate IGF-II expression in vertebrate development to some extent [34]. Later, in the larval stage and in the adult, ILP could play a function in endodermal tissues that recapitulates both vertebrate insulin and IGFs function. Interestingly, some studies suggest that amphioxus ILP displays growth factor activity in heterologous fish and mammalian cells but that it cannot reduce blood glucose level. This suggests that amphioxus ILP could be functionally closer to IGFs [15,35]. According to this view, insulins and IGFs would be derived paralogues that experience neofunctionalization after the duplication of a unique ILP ancestor protein in the vertebrate ancestor. This inference should however take into account that the amphioxus sequence is not an ancestral one but just the best available proxy and that amphioxus-specific events may have occurred. Nevertheless, additional functional studies are necessary to understand the ILP function in amphioxus.

## Conclusion

In this study, we identified a new ILP sequence in the European amphioxus *B*. *lanceolatum*. We analysed the phylogenetic position of ILP protein sequences that have been identified in different amphioxus species and conclude they form a monophyletic group that is the sister group of vertebrates insulins and IGFs. Moreover we characterized the spatio-temporal expression pattern of *ilp* in *B*. *lanceolatum* showing that its expression starts at early development and then is mainly expressed in the developing and adult gut.

Our phylogenetic and developmental analysis of ILP in amphioxus provides more clues in favour of a unique insulin/IGF protein in the vertebrate ancestor.

## Experimental Procedures

### Cloning

The *ilp* mRNA sequence from *B*. *californiensis* [3] was used as a template to search corresponding hits on *B*. *lanceolatum* reference transcriptome [19]. *In silico* reconstruction of *ilp* mRNA from *B*. *lanceolatum* allowed us to design specific primers for PCR amplification of a cDNA including the complete ILP coding sequence (Forward: GCATGAATCTATCCAGCGTG; Reverse: TCGGCTCAGTTGAGTGATAG).

Total RNA from adult *B*. *lanceolatum* was used as a template for reverse-transcription (Superscript III Invitrogen using random primers). Amplification was performed using Gold taq (Roche). Amplified fragments were cloned using the pGEM-TEasy system (Promega) and sequenced. A clone containing the full length *ilp* ORF (936bp) from *B*. *lanceolatum* was selected.

### Phylogenetic analysis

Protein sequences were obtained from Uniprot (http://www.uniprot.org/) or JGI website for *B*. *floridae* genome (http://genome.jgi-psf.org/Brafl1/Brafl1.home.html). The 35 sequence IDs of ILPs/insulins/IGFs/relaxins used for the phylogenetic analysis are reported in Table 1. Multiple sequence alignments were generated (ClustalW, MUSCLE, MAFFT) and analysed using Guidance [36]. The alignment with the best score was selected for further analysis (ClustalW, Fig. 1B). The alignment was manually corrected to avoid obvious inconsistencies due to the relatively low level of sequence conservation between the various ILPs. We checked that these changes did not result in major changes in the tree topology. Only the sites with the best alignment scores that correspond to B and A domains were used to generate the phylogenetic tree, the C, D and E peptides were omitted from the analysis. ProtTest [37] was used to establish the best-fitting model of amino acid substitution (LG). The phylogeny presented Fig. 1C was generated using the web interface http://www.atgc-montpellier.fr/ The tree was obtained with PhyML [38] using a LG model with 1000 bootstrap repetitions The relaxin sequences were used as the most appropriate available outgroup.

**Table 1:** Sequence IDs of members of the insulin-relaxin superfamily used to build the phylogeny depicted in Fig. 1C.

**organisms**	**species**	**protein**	**sequence ID**	**database**
amphioxus	*Branchiostoma californiensis*	ILP	P22334	UniprotKB
amphioxus	*Branchiostoma belcheri*	IGF-like	B1A4F5	UniprotKB
amphioxus	*Branchiostoma floridae*	ILP	121099	JGI
amphioxus	*Branchiostoma floridae*	ILP-like	77763	JGI
amphioxus	*Branchiostoma floridae*	ILP-like	97394	JGI
amphioxus	*Branchiostoma floridae*	ILP-like	100967	JGI
amphioxus	*Branchiostoma floridae*	ILP-like	72897	JGI
amphioxus	*Branchiostoma floridae*	ILP-like	74371	JGI
sea urchin	*Strongylocentrotus purpuratus*	ILP1	W4XUU8	UniprotKB
sea urchin	*Strongylocentrotus purpuratus*	ILP2	W4ZLE2	UniprotKB
human	*Homo sapiens*	insulin	P01308	UniprotKB
human	*Homo sapiens*	IGFI	P05019	UniprotKB
human	*Homo sapiens*	IGFII	P01344	UniprotKB
human	*Homo sapiens*	RLN3	Q8WXF3	UniprotKB
mouse	*Mus musculus*	insulin 1	P01325	UniprotKB
mouse	*Mus musculus*	insulin 2	P01326	UniprotKB
mouse	*Mus musculus*	IGFI	P05017	UniprotKB
mouse	*Mus musculus*	IGFII	P09535	UniprotKB
mouse	*Mus musculus*	RLN3	Q8CHK2	UniprotKB
chick	*Gallus gallus*	insulin	P67970	UniprotKB
chick	*Gallus gallus*	IGFI	P18254	UniprotKB
chick	*Gallus gallus*	IGFII	P33717	UniprotKB
chick	*Gallus gallus*	RLN3	B1AC67	UniprotKB
zebrafish	*Danio rerio*	insulin a	O73727	UniprotKB
zebrafish	*Danio rerio*	insulin b	Q2QAJ9	UniprotKB
zebrafish	*Danio rerio*	IGFI	Q90VV9	UniprotKB
zebrafish	*Danio rerio*	IGFIIa	Q5U3B4	UniprotKB
zebrafish	*Danio rerio*	IGFIIb	Q8JIE4	UniprotKB
zebrafish	*Danio rerio*	RLN3a	Q2VT45	UniprotKB
xenopus	*Xenopus laevis*	Insulin1	P12706	UniprotKB
xenopus	*Xenopus laevis*	Insulin2	P12707	UniprotKB
xenopus	*Xenopus laevis*	IGFIA	P16501	UniprotKB
xenopus	*Xenopus laevis*	IGFIIB	Q6INW9	UniprotKB
xenopus	*Xenopus laevis*	RLN3	B1PT68	UniprotKB

### Animal collection and spawning

European amphioxus *Branchiostoma lanceolatum* were collected (ripe adults) near Argelès-sur-Mer, France, (latitude 42° 32’ 53” N and longitude 3° 03’ 27” E) with a specific permission delivered by the Prefect of Region Provence Alpes Côte d’Azur. *Branchiostoma lanceolatum* is not a protected species. Animals were maintained in an artificial sea water at 19°C under natural light–dark conditions [39–41]. Spawning was induced with a thermal shock (from 19°C to 23°C) as previously described [39,40]. *In vitro* fertilizations were performed and synchronized embryos were collected at different developmental stages.

### Whole mount *in situ* hybridization

The plasmid containing the *ilp* cDNA from *B*. *lanceolatum* described in the “cloning” section was linearized with appropriate enzymes for sense and antisense probe synthesis and purified with sodium acetate precipitation. Linearized plasmids were then used as templates for synthesizing approximately 1 kb riboprobes using the DIG labelling system following manufacturer’s instructions (Roche). DIG-labelled riboprobes were purified using lithium chloride precipitation, dissolved in 50% formamide at 100 ng/μL, checked on an agarose gel and stored at −20°C

Embryos and larvae were collected as described in the “animal collection and spawning” section and fixed overnight at 4°C in 4% paraformaldehyde (PFA) in 0,1 M MOPS, 2 mM MgSO4, 1 mM EGTA, 0,5 M NaCl, pH 7,5. Fixed embryos and larvae were then washed and stored in 70% ethanol at −20°C.

The *in situ* hybridization protocol has been adapted from Holland et al., 1992 [42]. The different steps of the protocol were performed at room temperature (approximately 25°C), except when specified.

After 3 washes in NaPBSTw (20 mM NaPO4 buffer, 0.9% NaCl, 0.1% Tween 20), embryos and larvae were fixed for 1 hour in 4% PFA in NaPBSTw. After two washes in 0.1 M triethanolamine pH 8.0 (1 and 5 minutes, respectively), embryos and larvae were acetylated with two successive washes in 0.25% and 0,5% acetic anhydride in 0.1 M triethanolamine (5 minutes each), and then washed twice in NaPBSTw (1 and 5 minutes). Embryos and larvae were then washed and incubated 1 hour at 60°C in a prewarmed hybridization solution (50% formamide, 100 μg/ml heparin, 0,1% Tween 20 pH 7,5, 5X SSC, 5 mM EDTA, 1X Denhardt’s solution, 1 mg/ml yeast tRNA).

DIG-labelled riboprobes were diluted at 1 ng/μl in the hybridization buffer and incubated 5 minutes at 70°C. Hybridization was performed overnight at 60°C under agitation. After hybridization, embryos and larvae were washed at 60°C with prewarmed wash solution 1 (50% formamide, 5 X SSC, 1% SDS) (twice 5 minutes and twice 15 minutes), and with wash solution 2 (50% formamide, 2 X SSC, 1% SDS) (5 minutes), then cooled for 10 minutes at room temperature, washed again 15 minutes in wash solution 2 and washed twice with solution 3 (2 X SSC, 0,1% Tween 20) (1 and 5 minutes).

Embryos and larvae were then treated 20 minutes at 37°C with 10 μg/ml RNAase A and 10 U/ml RNAase T1 in wash solution 3. Treated embryos and larvae were then washed successively with wash solution 3 (twice 20 minutes), with wash solution 4 (0.2% SSC, 0.1% Tween 20) (20 minutes) and wash solution 5 (0.1% Tween 20, 2 mg/ml BSA in NaPBSTw) (5 minutes).

For immunological detection of the DIG label, embryos and larvae were incubated 1 hour with blocking solution (10% sheep serum in wash solution 5) before overnight incubation at 4°C with 1:3000 alkaline-phosphatase-conjugated anti-DIG (Roche) in wash solution 5.

Embryos and larvae were washed (4 times, 20 minutes) in NaPBSTw, then washed twice (1 and 5 minutes) with alkaline phosphatase buffer 1 (100 mM NaCl, 100 mM Tris-HCl pH 9.6, 0.1% Tween 20) and washed (3 times, 10 minutes) with alkaline phosphatase buffer 2 (100 mM NaCl, 50 mM MgCl2, 100 mM Tris-HCl pH 9.6, 0.1% Tween 20) before signal detection using BMPurple following the suppliers instructions (Roche). Reactions were performed in a black box until the signal appeared, and stopped in NaPBS. Labelled embryos and larvae were post fixed 1 hour with 4% PFA, washed twice with NaPBS and transferred into 80% glycerol in NaPBS. Embryos and larvae were mounted on slides and observed with Nomarski optics.

### qPCR analysis

Synchronized embryos obtained as described in the “animal collection and spawning” section were collected at different stages, frozen in liquid nitrogen and conserved at −80°C. Adult amphioxus were dissected and samples (anterior part, mid body, posterior part, muscle, gut and male and female gonads) were conserved in RNAlater (Qiagen) at −20°C. Precellys homogenizer (Ozyme) was used and total RNAs were extracted using RNeasy kit (Qiagen) following manufacturer’s instructions. Purity of extracted RNAs was analysed using Dropsense spectrophotometer (Trinean). RNAs were then treated with TurboDNAse (Ambion) following manufacturer’s instructions. Concentration of DNAse treated RNAs was analysed with Nanodrop spectrophotometer (Thermo Scientific) and quality of RNA samples was analysed using Tapestation (Agilent). Because of rarity of samples, only one RNA extraction has been performed for each condition (n = 1). Only RNAs with RINe>7,8 and concentration high enough for the reverse transcription of 200 ng of RNA for the analysis of *ilp* expression through development, or 500 ng of RNA for the analysis of *ilp* expression in the adult have been used in this study.

RNAs were reverse transcribed with High Capacity RNA-to-cDNA reverse transcriptase (Applied Biosystems) primed with a mixture of random hexamers and oligodT primers following manufacturer’s instructions.

Primers were designed using Primer3Plus (http://www.bioinformatics.nl/cgi-bin/primer3plus/primer3plus.cgi/) with qPCR settings. Primer sequences used in this study are reported on Table 2.

**Table 2:** Sequence of the primers used in the qPCR analysis.

Target gene	Sequence ID of homologous genes in the *B*. *floridae* genome (JGI)	Primer sequences	Primer efficiency	BestKeeper standard deviation in quantification through development	BestKeeper standard deviation in quantification in the adult
NR1H-2	Bf_124680	Fwd: CGCCCTATTGTTATGGAAGC, Rev: AAGTGATGCGGTGTTCTGC	1,961		0,66
NR1H-5	Bf_128090	Fwd: ATTTTGCCAGGCACGATG, Rev: CACTTGGCAAACTGAACGAG	1,891	0,68	
NR1H-6	Bf_124948	Fwd: TGCAGTGTGTGATAGTGGTGTC, Rev: GTAGGCGACTTTGCAGTAAGC	1,878	0,65	
NR1H-7	Bf_156544	Fwd: AGTCAAGGGTTCAGTTGTAGAGG, Rev: TATGGGCTGAGATGAGGATG	1,928	0,49	
NR1H-8	Bf_222287	Fwd: ACACCGCTTATGCAATCCTG, Rev: ATCGTCGTCTTGATGTACGC	1,925		0,45
EF1	Bf_58879	Fwd: GGAAGTTCGAGACCACCAAG, Rev: CACAATCAGCACAGCACAGTC	1,946		0,65
ILP	Bf_121099	Fwd: TCGGCTCCTTCGAAGATAAG, Rev: CGTTGGTTGGTCCTTCTTCTAC	1,877	2,1	1,87

qPCR reactions were performed following manufacturer’s instructions with IQ SYBR Green Supermix (Biorad) on CFX 96 Real-Time PCR Detection System (Biorad). The reaction conditions were as follows: 95°C for 3 min, followed by 45 cycles of 95°C for 10 s and 60°C for 30 s. Data were collected and curves were generated with CFX Manager software (Biorad). Each reaction (each condition with each primer couple) was carried out in technical triplicates. Dissociation analysis was performed at the end of each reaction to confirm the amplification specificity. On each reaction, a reverse transcriptase negative control was performed to test genomic DNA contamination.

In order to determine primer efficiencies, a template qPCR reaction was performed from adult cDNA for each primer couple and then diluted (7 dilution points) to generate linear standard curves. Primer efficiencies used in this study are reported in Table 2.

Genes coding nuclear receptors orthologous to vertebrates FXL/LXR (NR1H-1 to 10) [43] were analysed in parallel for another study on the same experimental conditions (same cDNAs) as for *ilp*, some of them appeared to be stable through development or in the adult. Stable genes were analysed using BestKeeper analysis [44] and used for normalization of *ilp* expression. The quantification of *ilp* expression through development was normalized with NR1H-5, NR1H-6 and NR1H-7 and the quantification of *ilp* expression in the adult was normalized with NR1H-2, NR1H-8 and with the elongation factor 1-alpha (EF1α) [45].

For each technical triplicate, the mean of Cq was calculated. For each condition, the gene expression level was calculated using primer efficiencies following the mathematical model of Pfaffl [46]. Then for each condition, the quantity of ILP was normalized with the geometrical mean of the values calculated for the three reference genes chosen for the analysis [47]. The relative quantities of ILP were then reported on graphs Fig. 2.

## Supporting Information

S1 Fig Phylogenetic relationships between amphioxus ILP and insulin/IGF proteins in chordates. Phylogenetic maximum likelihood analysis of chordates insulin/IGF family members using some relaxin sequences as outgroup. Bootstrap values derived from 1000 runs are shown. The scale bar indicates the average number of amino acid substitutions per site.
(TIF)

S2 Fig *In situ* hybridization control experiment. ISH of amphioxus embryos and larva obtained with the *ilp* control probe (sense probe). (A) Eight cells embryo. (B) Blastula stage embryo. (C) Dorsal view of a gastrula stage embryo. (D) Dorsal and (E) left side views of an early neurula embryo. (F) Left side view of a mid-neurula stage embryo. (G) Left side of a late neurula stage embryo. (H) Left side of the larvae.
(TIF)
