# Supplementary figures and images for: Identification, Evolution and Expression of an Insulin-Like Peptide in the Cephalochordate Branchiostoma lanceolatum
Source: PLoS One. 2015 Mar 16;10(3):e0119461. doi: 10.1371/journal.pone.0119461 (PMC4361685; doi:10.1371/journal.pone.0119461)

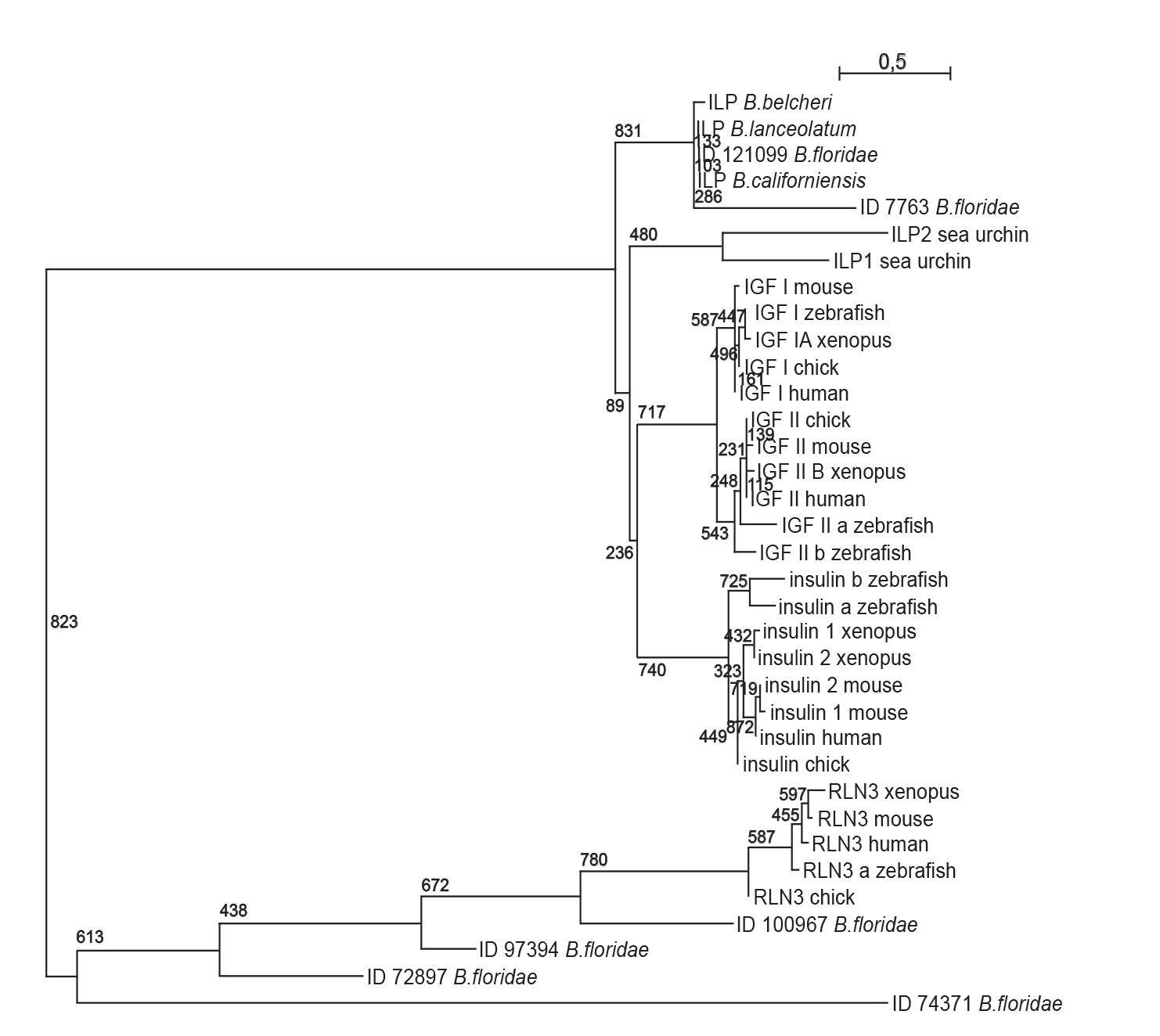

Supplement: S1 Fig — Phylogenetic maximum likelihood analysis of chordates insulin/IGF family members using some relaxin sequences as outgroup. Bootstrap values derived from 1000 runs are shown. The scale bar indicates the average number of amino acid substitutions per site. (TIF) [file pone.0119461.s001.tif]

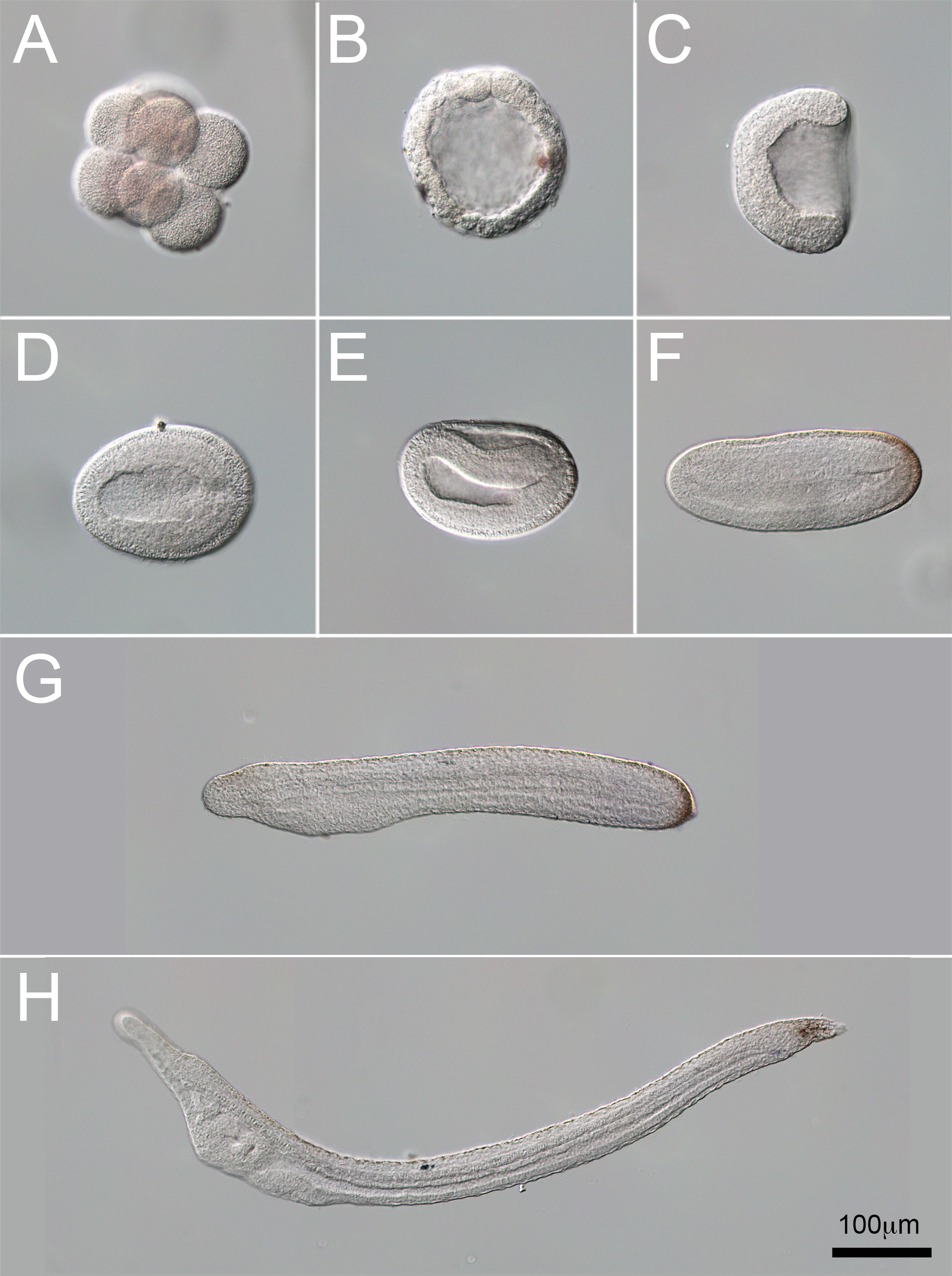

Supplement: S2 Fig — ISH of amphioxus embryos and larva obtained with the ilp control probe (sense probe). (A) Eight cells embryo. (B) Blastula stage embryo. (C) Dorsal view of a gastrula stage embryo. (D) Dorsal and (E) left side views of an early neurula embryo. (F) Left side view of a mid-neurula stage embryo. (G) Left side of a late neurula stage embryo. (H) Left side of the larvae. (TIF) [file pone.0119461.s002.tif]
